# Lipoglycans Contribute to Innate Immune Detection of Mycobacteria

**DOI:** 10.1371/journal.pone.0028476

**Published:** 2011-12-02

**Authors:** Shyam Krishna, Aurélie Ray, Shiv K. Dubey, Gérald Larrouy-Maumus, Christian Chalut, Romain Castanier, Audrey Noguera, Martine Gilleron, Germain Puzo, Alain Vercellone, K. Madhavan Nampoothiri, Jérôme Nigou

**Affiliations:** 1 CNRS, IPBS (Institut de Pharmacologie et de Biologie Structurale), Toulouse, France; 2 Université de Toulouse, UPS, IPBS, Toulouse, France; 3 Biotechnology Division, National Institute for Interdisciplinary Science and Technology, CSIR, Thiruvananthapuram, India; University of Maryland, United States of America

## Abstract

Innate immune recognition is based on the detection, by pattern recognition receptors (PRRs), of molecular structures that are unique to microorganisms. Lipoglycans are macromolecules specific to the cell envelope of mycobacteria and related genera. They have been described to be ligands, as purified molecules, of several PRRs, including the C-type lectins Mannose Receptor and DC-SIGN, as well as TLR2. However, whether they are really sensed by these receptors in the context of a bacterium infection remains unclear. To address this question, we used the model organism *Mycobacterium smegmatis* to generate mutants altered for the production of lipoglycans. Since their biosynthesis cannot be fully abrogated, we manipulated the biosynthesis pathway of GDP-Mannose to obtain some strains with either augmented (∼1.7 fold) or reduced (∼2 fold) production of lipoglycans. Interestingly, infection experiments demonstrated a direct correlation between the amount of lipoglycans in the bacterial cell envelope on one hand and the magnitude of innate immune signaling in TLR2 reporter cells, monocyte/macrophage THP-1 cell line and human dendritic cells, as revealed by NF-κB activation and IL-8 production, on the other hand. These data establish that lipoglycans are *bona fide* Microbe-Associated Molecular Patterns contributing to innate immune detection of mycobacteria, *via* TLR2 among other PRRs.

## Introduction

Innate immune recognition is based on the detection of molecular structures that are unique to microorganisms [1]. It involves a limited number of germline-encoded pattern recognition receptors (PRRs) that recognize conserved molecules of microbes, referred to as microbe-associated molecular patterns (MAMPs) [2]. MAMPs follow three criteria: i) they have an invariant core structure among a given class of microorganisms, ii) they are products of pathways that are unique to microorganisms and iii) they are essential for the survival of the microorganism and are therefore difficult for it to alter [1]. Most of them have been characterized by their capacity, as purified molecules, to bind PRRs and/or to activate PRR-mediated signaling. However, whether they really contribute to microbe recognition by innate immune system in a physiological context is not always clearly demonstrated and remains for some of them under debate [3]. The most controversial situation is probably that observed for TLR2 ligands. Indeed, of all TLRs, TLR2 is the receptor that recognizes the structurally broadest range of MAMPs [3]. Its ligands are as diverse as lipoproteins, lipopeptides, lipoteichoic acid (LTA), peptidoglycan, zymosan, GPI anchors or lipoglycans [3]. This high diversity in ligand recognition has been proposed to possibly arise, at least in part, from its capacity to function as a heterodimer with either TLR1 or TLR6 [4]. However, because some of these molecules are structurally unrelated, their real nature as TLR2 ligands is a matter of controversy [3]. Indeed, no obvious structure-function relationship can be drawn as one could expect from an ordinary receptor-ligand interaction [5]. This chaotic situation results from both the use of incompletely defined agonist preparations and the lack, until very recently, of high resolution structural data defining these interactions at the atomic level [3]. For example, the TLR2 activity originally found in some commercially available LPS preparations was subsequently demonstrated to arise from endogenous contaminating lipoproteins [6], [7]. A similar explanation is advanced for the observed TLR2 activity in peptidoglycan fractions [3]. This assumption is reinforced by the recently published crystal structure of a TLR1-TLR2 heterodimer in complex with the model lipopeptide Pam_3_CSK_4_ [8]. Indeed, it clearly shows the importance of ligand acyl chains to bind and induce heterodimerization of the receptors and provides a rationale to tentatively understand the ligand structure-function relationships, although the presence of binding sites other than that of lipopeptides cannot be excluded [9], [10]. For instance, LTA, that bears two acyl chains, has been unambiguously proved, using chemically synthesized analogs, to stimulate TLR2 [11] and recently demonstrated to bind TLR2 [12]. However, its role as a physiological TLR2 ligand is still under debate [3], [13], [14]. Indeed, a set of studies focusing on *Staphylococcus aureus* and using cell wall-derived compounds as well as a mutant lacking acylated lipoproteins, demonstrates that LTA is much less active than lipoproteins and suggests that not LTA but lipoproteins are the dominant immunobiologically active compounds in this Gram-positive bacterium [3], [13]. As a consequence, in a recent review, Zähringer *et al* [3] propose that lipoproteins/lipopeptides are the only compounds of microorganisms sensed at physiological concentrations by TLR2.

Lipoglycans are surface-exposed molecules of mycobacteria [15], [16], [17] that have been described by other and us to be ligands, as purified molecules, of several PRRs, including the C-type lectins Mannose Receptor and DC-SIGN, as well as TLR2 (For a recent review, see [18]). However, their real nature as MAMPs has never been validated by isogenic mycobacterial mutants in the context of a bacterium infection. Their structure is based on a mannosyl-phosphatidyl-*myo*-inositol anchor, which, although very similar to the GPI anchors found in eukaryotic cells, is specific of these microorganisms [18]. The biosynthesis of the mannosyl-phosphatidyl-*myo*-inositol anchor is essential in mycobacteria [18], [19]. The most active lipoglycan, lipomannan (LM), is sensed by TLR2 at concentrations similar to that of mycobacterial lipoproteins and we have shown recently that it can compete for lipopeptide binding to the receptor, suggesting that it shares at least in part the same binding site [20]. Assuming that it is the case, straightforward structure-function relationships can account for the observed TLR2-stimulatory capacity of the various purified LM acyl-forms [20], [21]. Nevertheless, a contamination of lipoglycan fractions by highly active lipopeptides is formally difficult to rule out. Moreover, a *Mycobacterium tuberculosis* mutant deficient for lipoprotein processing is dramatically altered in its capacity to stimulate TLR2 [22], suggesting, as for *S. aureus*, a predominant role of lipoproteins in mycobacteria sensing by TLR2.

In order to determine whether lipoglycans are i) *bona fide* MAMPs and most particularly TLR2 ligands and ii) sensed at physiological concentrations in the context of the whole bacterium, we used here the model organism *Mycobacterium smegmatis* to generate mutants altered for the production of lipoglycans. Since their biosynthesis cannot be fully abrogated [19], we attempted to construct some strains with either an increased or a reduced production of lipoglycans and we compared their ability to induce innate immune signaling relatively to control strains in reporter cells, macrophage cell line or dendritic cells. Finally, to compare the relative contribution of lipoglycans and lipoproteins in mycobacteria sensing by TLR2, we constructed a mutant deficient for lipoprotein processing.

## Materials and Methods

### Construction of the *M. smegmatis* recombinant strains

*Rv3255c* (*manA*), *Rv3257c* (*manB*), *Rv3308* (*pmmB*) and *Rv3264c* (*manC*) genes were amplified from *M. tuberculosis* H37Rv genomic DNA (TB Vaccine Testing and Research Materials Contract, NIH, NIAID NO1-AI-40091) using the primers described in Figure S1. The resulting PCR products were purified and digested with HindIII on one hand and EcoRV (*Rv3255c* and *Rv3264c)*, Bal I (*Rv3257c)* and SmaI for (*Rv3308)* on the other hand. Restricted PCR products were ligated into the BalI and HindIII-digested pMV261 *E. coli*-mycobacteria shuttle vector [23], giving rise to the pMV*manA*, pMV*manC*, pMV*manB* and pMV*pmmB*. These plasmids were transferred into *M. smegmatis* mc^2^155 by electroporation.

The strains were grown at 37°C in Middlebrook 7H9 medium (Difco) supplemented with 40 µg/ml Kanamycin (Km).

### Construction of the *pmmB*- and *lspA*-disrupted mutants of *M. smegmatis*

A DNA fragment overlapping the *pmmB* gene (*MSMEG_1695*) was amplified by PCR from *M*. *smegmatis* mc^2^155 genomic DNA using primers FP and RP (Figure S1). This PCR fragment was cloned after insertion of a Km resistance gene surrounded by two *res* sites from transposon γδ (*res*-*km*-*res* cassette) between the EcoRI and NcoI restriction sites, into the suicide plasmid pJQ200 [24], [25]. This plasmid was electrotransferred into *M*. *smegmatis* and the transformants were selected on plates containing Km (40 µg/ml) and 5% sucrose. PCR screening for disruption of *pmmB* was performed with a set of specific primers (FBP, RPB, RPI, res1 and res2, Figure S1) after extraction of the genomic DNA from several Km- and sucrose resistant colonies. One clone giving the corresponding pattern for disruption of *pmmB* was selected for further analyses and named *Msmeg*/Δ*pmmB*.

To construct the *lspA*-disrupted mutant of *M. smegmatis*, a 1700 bp DNA fragment containing the *lspA* gene (*MSMEG_3174*) of *M. smegmatis* mc^2^155 was amplified by PCR from genomic DNA using oligonucleotides 3181A and 3181B (Figure S1). The PCR product was digested with NotI and cloned, after insertion of the *res*-*km*-*res* cassette at the NcoI restriction site, into the mycobacterial thermosensitive plasmid pCG217 which contains the counterselectable marker *sacB* and the *xylE* reporter gene. The resulting vector was electrotransformed in *M. smegmatis* mc^2^155, and transformants were selected on 7H11 agar plates containing Km (40 µg/ml) at 32°C. Few clones were selected and grown in 5 mL of 7H9 medium containing Km at 32°C for 3 days. Several dilutions of these cultures were then plated onto 7H11 agar plates containing Km, catechol and 2% sucrose, and incubated at a non-permissive temperature (42°C). PCR screening for disruption of *lspA* was performed with a set of specific primers (3181C, 3181D, res1b, res2b, kan1, kan2) (Figure S1) after extraction of the genomic DNA from several Km- and sucrose-resistant colonies. One clone giving the corresponding pattern for disruption of *lspA* was selected for further analyses and named *Msmeg*/Δ*lspA*.

To generate a *lspA* mutated strain carrying no antibiotic resistant marker, the *Msmeg*/Δ*lspA* mutant was transformed with the thermosensitive plasmid pWM19, which contains the resolvase gene of transposon γδ and an hygromycin (Hyg) resistance gene [24]. Transformants were resuspended in 5 ml of 7H9 medium and incubated for 6 h at 32°C to allow the expression of Hyg resistance. Hyg (100 µg/ml) was then added to the transformation mixture, and cells were incubated for 24 h at 32°C. Serial dilutions of the bacterial culture were then plated on 7H11 plates and incubated at 42°C. Several colonies were picked and tested for growth on plates containing Km. Two clones that were unable to grow on Km-containing plates but that showed normal growth on control antibiotic-free plates were selected and analyzed by PCR using primers 3181C and 3181D. One clone giving the corresponding pattern for excision of the Km resistance cassette in *lspA* was retained for further analysis and named *Msmeg*/Δl*spAres*.

### Construction of complementation plasmids

Complementation plasmids pMV/*MSMEG_1695* and pMV/*MSMEG_3174* were constructed by amplifying *pmmB* and *lspA* from *M. smegmatis* mc^2^155 genomic DNA using primers PMFP and PMRP and primers 3181I and 3181J respectively (Figure S1). The PCR products were digested with NdeI and HindIII and cloned between the NdeI and HindIII sites of pMV361eHyg, a pMV361 derivative containing the pblaF* promoter instead of the original *phsp60* promoter and carrying a *hyg* resistance marker [23]. pMV/*MSMEG_1695* was transferred into the *Msmeg*/Δ*pmmB* mutant strain and pMV/*MSMEG_3174* was transferred into the *Msmeg*/Δ*lspA* and *Msmeg*/Δ*lspAres* mutants. Transformants were selected on 7H11 agar plates supplemented with Hyg (50 µg/ml) and Km (40 µg/ml) when necessary.

### Expression of recombinant protein Msmeg1712-HA

The vector pHSG84 encoding the endogenous lipoprotein Msmeg1712-hemagglutinin (HA) tagged was graciously provided by Miriam Braunstein (University of North Carolina at Chapel Hill). Msmeg1712-HA was expressed from the native *MSMEG_1712* promoter. The plasmid pHSG84, carrying a Km resistance gene, was electrotransformed in *Msmeg*/Δ*lspAres* strain and its complemented counterpart. The transformants were selected on 7H11 agar plates containing kanamycin (40 µg/ml). Few clones were selected for Msmeg1712-HA expression analysis by western blot.

### Enzymatic assays

Recombinant strains were grown in the presence of 0.05% Tween 80 until OD at 600 nm reached 1.0, harvested by centrifugation, washed with PBS and disrupted by sonication. Cell lysates were centrifuged at 4°C for 30 min at 27000 g and the supernatants were used for enzyme assays. Protein concentration was determined using the Bio-Rad protein assay. Enzymatic activities were determined at 37°C in 1 ml reaction volume (4 mg of proteins) by monitoring the reduction of NADP to NADPH at 340 nm, using coupled enzyme reactions as previously described [26], [27], [28].

### Carbohydrate quantification and lipoglycan analysis by SDS-PAGE

Enriched lipoglycan fractions containing glycans and proteins were prepared as previously described [15]. Briefly, mycobacterial cells were delipidated by several extractions with CHCl_3_/CH_3_OH (1∶1, v/v), yielding a lipidic extract containing PIM. Delipidated cells were then disrupted by sonication. Lipoglycans, LM and LAM, were further extracted by refluxing the broken cells in 50% ethanol at 65°C. Contaminating nucleic acids and glucans were removed by enzymatic degradation treatments followed by dialysis. Proteins were degraded by protease digestion to avoid their superimposition with lipoglycans on the SDS-PAGE gel but were not removed from the fractions to keep them as an internal reference between the different strains. Arabinose and mannose were quantified from an equivalent amount of 1 µg of proteins by capillary electrophoresis after total acid hydrolysis [29]. Lipoglycans were analyzed by SDS-PAGE (10 µg of proteins were loaded on the gel for each strain) followed by periodic acid-silver nitrate staining.

### Lipoprotein quantification

Mycobacterial cells (1.5 g) were disrupted by sonication and unbroken cells were removed by gentle centrifugation. Lipoproteins were extracted from the cleared lysate by a phenol/water partition [6], [7]. The phenol phase containing lipoproteins was dialyzed against water, dried and weighed. No significant variation could be observed between the different strains.

### HEK-TLR2 experiments

HEK-Blue^TM^-2 cells (Invivogen, Toulouse, France), derivatives of HEK293 cells that stably express the human TLR2 and CD14 genes along with a NF-κB-inducible reporter system (secreted alkaline phosphatase) were added in the HEK-Blue^TM^ Detection medium (Invivogen), which contains a substrate for alkaline phosphatase, at 5×10^4^ cells per well in 96-wells plates and incubated with the different mycobacterial strains at MOI ranging from 1 to 0.1. Alkaline phosphatase activity was measured after 18 hours by reading O.D. at 630 nm.

### THP-1 experiments

THP-1-Blue^TM^ cells (Invivogen), derivatives of THP-1 monocyte/macrophage human cells that stably express a NF-κB-inducible reporter system (secreted alkaline phosphatase), were added at 10^5^ cells per well in 96-wells plates in HEK-Blue^TM^ Detection medium (for NF-κB activation assay) or differentiated with 20 ng/ml of PMA for 24 h in RPMI 1640 culture medium (Lonza, Verviers, Belgium) (for IL-8 production assay). The different mycobacterial strains were added at MOI ranging from 10 to 1 and, after 18 h, NF-κB activation was measured as described above or cytokines were assayed in the supernatant by sandwich ELISA using commercially available kits (Diaclone, Besançon, France). To investigate TLR2 dependence, cells were pre-incubated for 30 min at 37°C, before bacteria addition, with 5 µg/ml of anti-TLR2 antibodies (IgG1 clone T2.5, IgG2a clone TL2.1 or IgA1, Invivogen) or isotype controls (IgG1, IgG2a, eBioscience or IgA1, Invivogen). To determine bacilli uptake, PMA-treated cells were infected for 1 h at 37°C with the various *M. smegmatis* strains at MOI of 50, extensively washed, lysed and plated onto agar for CFU counting.

### Dendritic cell experiments

Monocytes were obtained from healthy blood donors (Etablissement Français du Sang, EFS, Toulouse). Written informed consents were obtained from the donors under EFS contract nu21/PVNT/TOU/IPBS01/2009–0052. Following articles L1243-4 and R1243-61 of the French Public Health Code, the contract was approved by the French Ministry of Science and Technology (agreement n°AC 2009–921). Ethical approval was not required in this case as only fluids in surplus were used in this study, according to institutional guidelines.

Peripheral blood mononuclear cells were purified by Ficoll-Paque centrifugation. Monocytes were purified by positive selection using anti-CD14-coated magnetic microbeads (Miltenyi Biotec). Sorted cells were >98% CD14+ as assessed by flow cytometry staining. The recovered cells, referred to as monocytes, were seeded in 6-well plates at 2×10^6^ cells/well in 1.5 ml DC-medium described as RPMI 1640, 10% FCS (Lonza, Verviers, Belgium) supplemented with 800 UI/ml GM-CSF and 500 UI/ml IL-4 (Peprotech France). 0.5 ml fresh DC-medium was added to culture at day 2. At day 5, cells were resuspended in fresh medium and the different mycobacterial strains were added at a MOI of 1. After 18 h, cytokines were assayed in the supernatant by sandwich ELISA using commercially available kits (Diaclone, Besançon, France). For monitoring CD40 and CD86 expression, cells were harvested and resuspended in Dubelco's PBS, 0.5% BSA and labelled with CD40- or CD86-Phycoerythrin conjugated antibody (Beckman Coulter). Cells were subjected to flow cytometry analysis by using the CellQuest software on a flow cytometer (FACSCalibur, Becton Dickinson).

### Statistical analysis

Results are expressed as a mean ± SD and were analyzed using One-way analysis of variance followed by Tukey test to determine significant differences between samples.

## Results and Discussion

### Construction of *M. smegmatis* strains with an increased production of lipoglycans

**Figure 1:**
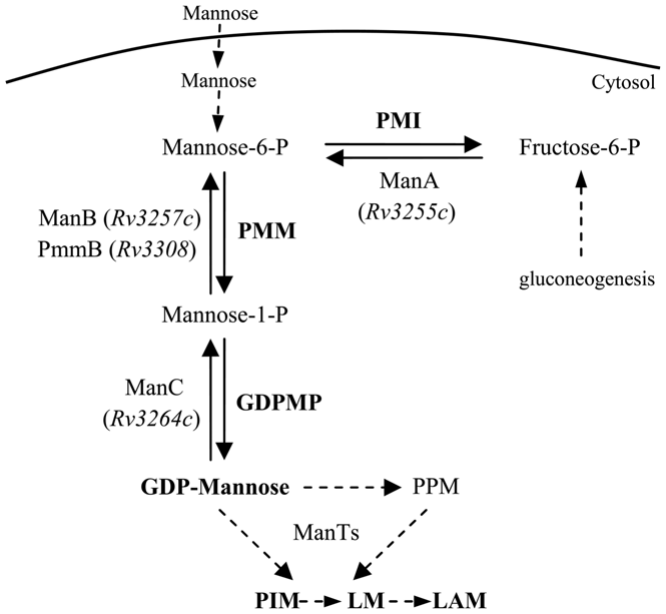
GDP-Mannose biosynthesis pathway in mycobacteria. Enzymatic steps considered in the present study are indicated with plain line arrows. ManTs, mannosyltransferases; GDPMP, GDP-Mannose pyrophosphorylase; PMI, phosphomannose isomerase; PMM, phosphomannomutase; PPM, polyprenyl-monophosphoryl-mannose.

**Figure 2:**
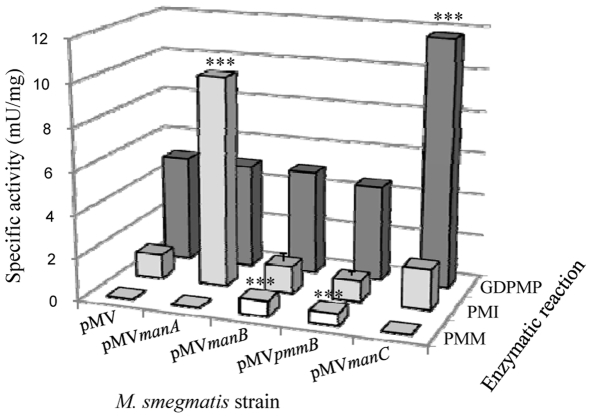
PMI, PMM and GDPMP enzymatic activities in the *M. smegmatis* recombinant strains. Enzymatic activities were determined on bacterial lysates using coupled assays [26], [27], [28]. The results are presented as mU enzyme activity per mg protein of the lysate and are mean ± SD of three separate experiments using independent bacterial cultures. ***, P<0.001.

**Figure 3:**
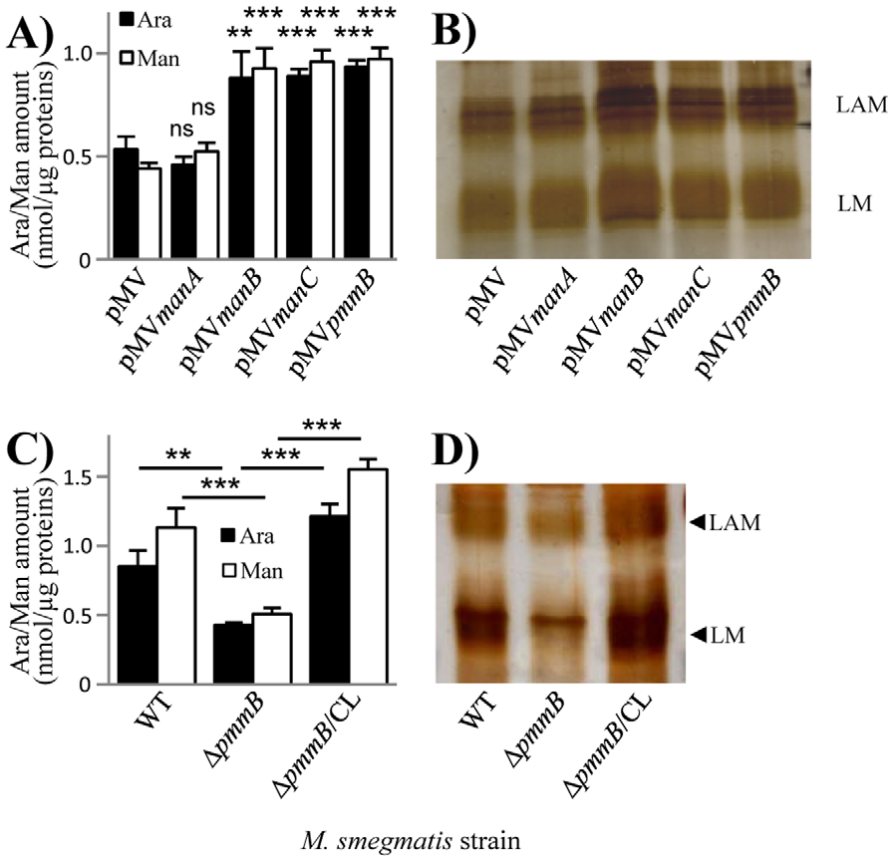
Altering the GDP-Mannose biosynthesis pathway impacts lipoglycan production. A, C) Arabinose and mannose quantification in a lipoglycan-enriched fraction. These monosaccharides were quantified from an equivalent amount of 1 µg of proteins by capillary electrophoresis after total acid hydrolysis [29]. The results are presented as nmol of the monosaccharide per µg protein in the fraction and are mean ± SD of triplicate assays from a representative experiment. Ara, arabinose; Man, mannose. **, P<0.01; ***, P<0.001; ns, not significant. In A), P value is given *vs* pMV. B, D) Lipoglycan analysis by SDS-PAGE and silver nitrate staining. Lipoglycans were loaded on the gel on a protein basis. A representative gel is shown.

Lipoglycans are biosynthesized *via* the sequential addition by mannosyltransferases (ManTs) of mannosyl units on phosphatidyl-*myo*-inositol, yielding phosphatidyl-*myo*-inositol mannosides (PIM) and LM (Figure 1) [18]. LM can be further arabinosylated to give lipoarabinomannan (LAM). ManTs involved in the first mannosylation steps use the soluble sugar nucleotide GDP-Mannose as mannose donor whereas those catalyzing later steps require the polyprenyl-phosphate based sugar donor, polyprenyl-monophosphoryl-mannose. However the latter derives from GDP-Mannose, which is thus the primary mannose donor in mycobacteria [30]. GDP-Mannose can be produced in three steps from either exogenously acquired mannose or gluconeogenesis-derived fructose-6-phosphate. Mannose and fructose-6-phosphate are converted to mannose-6-phosphate by the action of a hexokinase and a phosphomannose isomerase (PMI), respectively. Then mannose-6-phosphate is transformed to mannose-1-phosphate by a phosphomannomutase (PMM) and finally to GDP-Mannose by a GDP-Mannose pyrophosphorylase (GDPMP) (Figure 1). *Rv3255c*, *Rv3257c* and *Rv3264c* have been shown to encode PMI [31], PMM [32] and GDPMP [33] enzymes respectively and accordingly have been renamed *manA*, *manB* and *manC* [32], [34]. *manA* was proved to be essential for mycobacterial growth *in vitro* in the absence of an exogenous source of mannose [31]. High density mutagenesis predicted *manB* and *manC* to be also essential in *M. tuberculosis* [35]. In this context, we reasoned that boosting the GDP-Mannose pathway in mycobacteria could result in a subsequent increased production of lipoglycans. The proof of that concept was demonstrated by McCarthy *et al.* [32] who showed that *M. smegmatis* overexpressing *M. tuberculosis manB* gene produced increased levels of PIM, LM and LAM. We thus PCR amplified *manA*, *manB*, *manC* and *pmmB* gene copies from *M. tuberculosis* H37Rv [36] and inserted them separately in the *E. coli*-*Mycobacterium* shuttle vector pMV261. *pmmB* putative gene product shows some homology with known PMMs [32], [36]. The resulting constructs, and the control plasmid carrying no insert gene, were used to transform *M. smegmatis* mc^2^155. We then assessed PMI, PMM and GDPMP enzymatic activities in the different recombinant strains using *in vitro* assays on bacterial lysates. As expected, overexpression of *manA* resulted in a more than 6-fold increase in PMI activity as compared to the control strain whereas PMM and GDPMP activities were not affected (Figure 2). Similarly, GDPMP activity was augmented by around 2.5-fold in the *manC* overexpressing strain (*Msmeg*/pMV*manC*) but not in the other ones (Figure 2). In the conditions used, no PMM activity could be detected in the control or non-relevant strains. However, an activity of around 0.6 mU/mg of total proteins was measured in both strains overexpressing *manB* (*Msmeg*/pMV*manB*) or *pmmB* (*Msmeg*/pMV*pmmB*) (Figure 2), indicating that PmmB is an additional mycobacterial PMM. We thus had a set of recombinant strains showing an individual increase of each enzymatic activity involved in GDP-mannose biosynthesis. We then examined the consequences in term of lipoglycan production. In a first approach, we quantified in a lipoglycan-enriched fraction the amount of the carbohydrates mannose and arabinose, known to compose lipoglycans. An increase of around 1.7 fold of these carbohydrates was observed for *Msmeg*/pMV*manB*, *Msmeg*/pMV*pmmB* and *Msmeg*/pMV*manC* as compared to the control strain (*Msmeg*/pMV) whereas no change was detected in *Msmeg*/pMV*manA* (Figure 3A). These results suggested an increase of lipoglycan production in *Msmeg*/pMV*manB*, *Msmeg*/pMV*pmmB* and *Msmeg*/pMV*manC* strains. We further analyzed lipoglycans by SDS-PAGE followed by silver nitrate staining. They were loaded on the gel according to a fixed amount of proteins. The gels showed the presence of a higher amount of lipoglycans, with a slight increase in the apparent molecular weight, in the same three strains (Figure 3B). In contrast, we could not detect any significant difference in the content of PIM among the various strains, as revealed by thin-layer chromatography and MALDI mass spectrometry analyses of the lipidic extracts (not shown). These data indicate that the GDP-Mannose pool is in default amount for LM and LAM biosynthesis in *M. smegmatis*, essentially as a result of a limiting PMM activity.

### Construction of a *M. smegmatis* strain with a reduced production of lipoglycans

We have demonstrated above that *pmmB* gene product encodes an enzyme with PMM activity and that the latter affects lipoglycan biosynthesis (Figures 2, 3A and 3B). High density mutagenesis previously proved that this gene was non-essential in *M. tuberculosis* [35]. We thus investigated whether the deletion of its ortholog in *M. smegmatis* (*MSMEG_1695*) could result in a strain producing reduced amount of lipoglycans. We constructed a *M. smegmatis* mutant by exchanging the wild-type allele of *MSMEG_1695* with a Km resistance cassette-disrupted allele. One clone, named *Msmeg*/Δ*pmmB*, exhibiting an amplification pattern consistent with an allelic exchange at the *MSMEG_1695* locus, was retained for further analyses. Carbohydrate quantification, as performed before, showed that the lipoglycan-enriched fraction of *Msmeg*/Δ*pmmB* contained only half amount of arabinose and mannose as compared to the wild-type strain (Figure 3C). SDS-PAGE analysis confirmed that *Msmeg*/Δ*pmmB* produced reduced amount of LAM and LM as compared to the wild-type strain (Figure 3D). Lipoglycan production was restored in the mutant upon complemention with pMV/*MSMEG_1695* (*Msmeg*/Δ*pmmB*/CL). Again the production of PIM was not affected in the mutant strain (not shown).

### Consequences of lipoglycan altered production on mycobacteria-induced innate immune signaling

**Figure 4:**
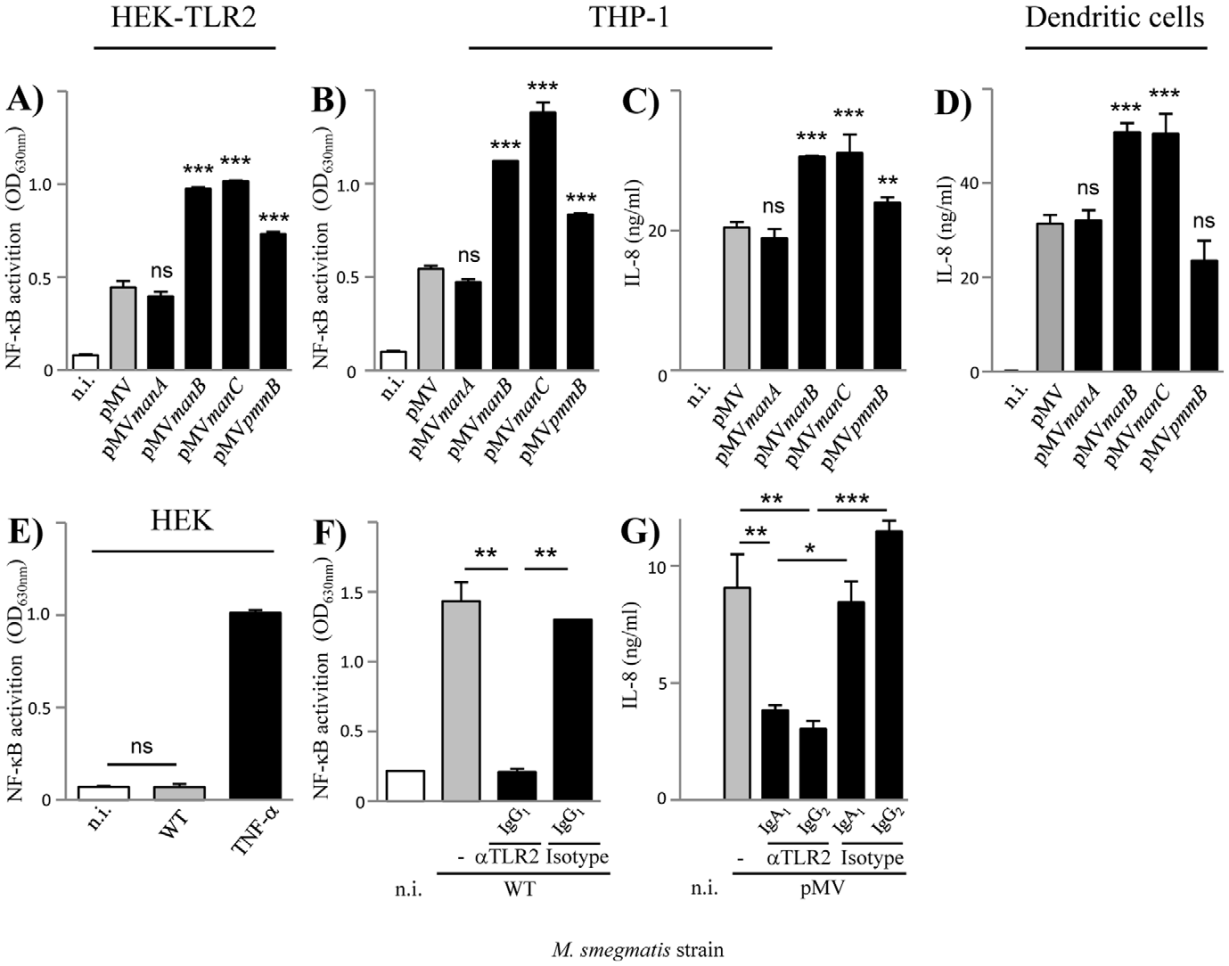
Lipoglycan increased production is associated with an enhancement of innate immune signaling. A, B, E, F) NF-κB activation in HEK (A, E) and THP-1 (B, F) cells. C, D, G) IL-8 production by THP-1 (C, G) and human monocyte-derived dendritic (D) cells. Cells were incubated with bacteria at MOI of 1. In F and G, cells were pre-incubated for 30 min at 37°C before bacteria addition with various antibodies, anti-TLR2 and isotype controls, at a concentration of 5 µg/ml. The results are mean ± SD of triplicate wells and are representative of at least three separate experiments using independent bacterial cultures and different MOI. *, P<0.05; **, P<0.01; ***, P<0.001; ns, not significant. In A, B, C and D), P value is given *vs* pMV. n.i., not induced.

**Figure 5:**
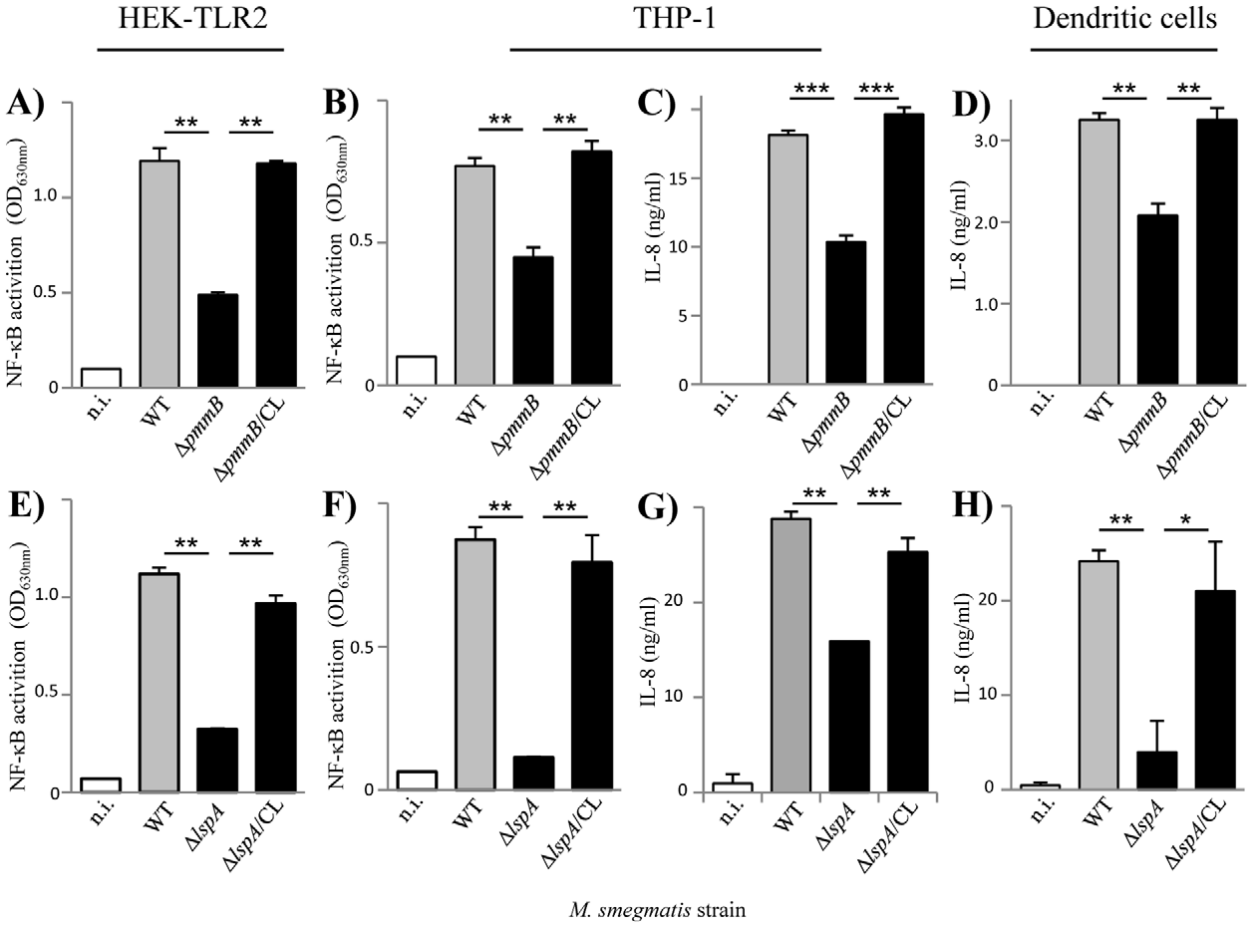
Lipoglycan reduced production is associated with a decrease of innate immune signaling. A, B, E, F) NF-κB activation in HEK-TLR2 (A, E) and THP-1 (B, F) cells. C, D, G, H) IL-8 production by THP-1 (C, G) and human monocyte-derived dendritic (D, H) cells. Cells were incubated with bacteria at MOI of 1. The results are mean ± SD of triplicate wells and are representative of at least three separate experiments using independent bacterial cultures and different MOI. *, P<0.05; **, P<0.01; ***, P<0.001. n.i., not induced.

Three *M. smegmatis* recombinant strains moderately overproducing lipoglycans, *Msmeg*/pMV*manB*, *Msmeg*/pMV*pmmB* and *Msmeg*/pMV*manC*, and a knock-out mutant with a reduced production of lipoglycans, *Msmeg*/Δ*pmmB*, were thus available to evaluate the possible implication of these compounds in mycobacteria sensing by innate immune receptors, including TLR2. It is noteworthy that these strains contained wild-type amount of lipoproteins (55±3 mg per 1.5 g cells) as determined by analysis and quantification of the phenol extracts. We first tested the relative ability of the different strains to stimulate HEK293 cells stably transfected with human TLR2 and CD14 genes (HEK-TLR2 cells) and a NF-κB-inducible reporter system. All the strains induced NF-κB activation in HEK-TLR2 cells (Figures 4A and 5A) but not in the parent HEK cells (Figure 4E and data not shown), demonstrating that activation was specific for TLR2. However, *Msmeg*/pMV*manB*, *Msmeg*/pMV*pmmB* and *Msmeg*/pMV*manC* were reproducibly more stimulatory than the control strains, *Msmeg*/pMV, and *Msmeg*/pMV*manA* (Figure 4A), whereas *Msmeg*/Δ*pmmB* was less stimulatory than the wild-type strain (Figure 5A), demonstrating an association between the level of lipoglycan production on one hand and the magnitude of TLR2 signaling on the other hand. To confirm it in more physiological cells, we investigated the relative capacity of the different strains to activate the human THP-1 monocyte/macrophage cell line, using a cell line derivative that stably expresses a NF-κB-inducible reporter system. Again, we found that *Msmeg*/pMV*manB*, *Msmeg*/pMV*pmmB* and *Msmeg*/pMV*manC* were more potent than the control strains in their ability to induce NF-κB activation (Figure 4B) and IL-8 (Figure 4C) and TNF-α (not shown) release. THP-1 cells being strong producers of IL-8, this chemokine was further used as readout of cell activation. Both NF-κB activation (Figure 4F) and IL-8 production (Figure 4G) were found to depend in part on TLR2 signaling, as determined by antibody blocking experiments. Interestingly, *Msmeg*/Δ*pmmB* was less potent than the wild-type strain in its ability to induce NF-κB activation (Figure 5B) and IL-8 release (Figure 5C) in THP-1 cells. But this defect did not impact on its uptake by these cells (Figure S2A). The different recombinant strains were finally tested for their ability to induce IL-8 production by and maturation of human monocyte-derived dendritic cells (DCs). *Msmeg*/pMV*manB* and *Msmeg*/pMV*manC* induced much more IL-8 than the control strains (Figure 4D) but only a slightly higher expression of CD86 and CD40 maturation markers (Figure S3). *Msmeg*/pMV*pmmB* stimulated IL-8 (Figure 4D) and CD86 and CD40 (Figure S3) to a level similar to the control strains, but *Msmeg*/Δ*pmmB* mutant was found again to be less stimulatory than the wild-type strain (Figure 5D).

Altogether the data demonstrate that lipoglycans are MAMPs contributing to innate immune detection of *M. smegmatis* by phagocytic cells, *via* TLR2 among other possible PRRs, as revealed by NF-κB activation and IL-8 production.

### Construction and characterization of *M. smegmatis lspA* mutant

**Figure 6:**
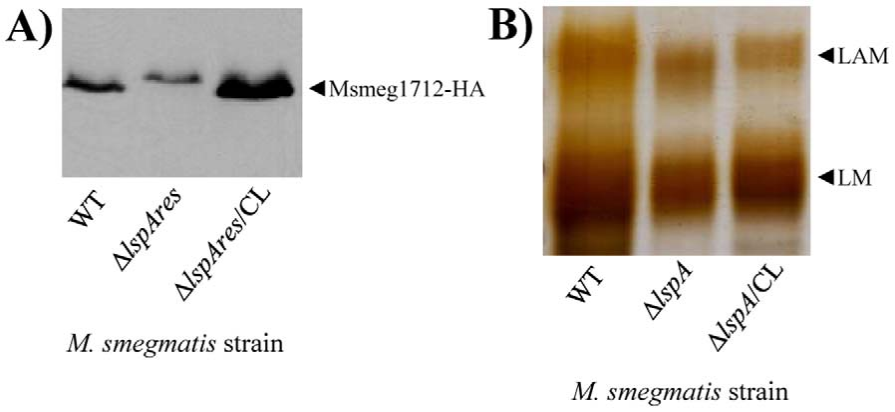
Phenotype of *M. smegmatis lspA* knock-out mutant. A) Western immunoblot of bacterial lysates with anti-HA antibody showing the migration of Msmeg1712-HA lipoprotein recombinantly expressed in *M. smegmatis* wild-type, in the unmarked *lspA* deletion mutant (*Msmeg*/Δ*lspAres*) and in the M*smeg*/Δ*lspAres* mutant complemented with plasmid pMV/*MSMEG_3174* (Δ*lspAres*/CL) strains. 10 µg of proteins were loaded. These strains were transformed with pHSG84 vector encoding HA-tagged endogenous lipoprotein Msmeg1712 [41]. B) Lipoglycan analysis by SDS-PAGE and silver nitrate staining. 5 µg of lipoglycans purified from *M. smegmatis* wild-type, Δ*lspA* mutant and Δ*lspA* mutant complemented with plasmid pMV/*MSMEG_3174* (Δ*lspA*/CL) strains were loaded.

As far as TLR2 is concerned, lipoproteins/lipopeptides are considered to be the strongest agonists of the receptor characterized so far [3]. Accordingly, an *M. tuberculosis* mutant deficient for lipoprotein processing (Δ*lspA*) was found to be impaired in its capacity to stimulate TLR2 in reporter cells [22], strongly suggesting that lipoproteins play a predominant role in mycobacteria sensing by TLR2 [37], [38]. To investigate the relative contribution of lipoproteins and lipoglycans in this process, we constructed a deletion mutant of *lspA* in *M. smegmatis* by allelic exchange, *Msmeg*/Δ*lspA*. *lspA* gene encodes prelipoprotein signal peptidase whose absence results in lipoproteins with a larger apparent molecular weight on SDS-PAGE, as a result of a non-cleaved off signal peptide [22], [39], [40]. Since very few *M. smegmatis* lipoproteins have been characterized so far and no antibody is available, an unmarked *lspA* deletion mutant, *Msmeg*/Δ*lspAres*, was transformed with a plasmid encoding the HA-tagged endogenous lipoprotein Msmeg1712 [41]. As expected, immunoblotting revealed that migration of Msmeg1712-HA was slower in *Msmeg*/Δ*lspAres* than that in the wild-type (Figure 6A). A wild-type migration was restored when the mutant strain was complemented with the wild-type allele of *lspA*. In agreement with the results of Banaiee *et al.* [22], *Msmeg*/Δ*lspA* showed a dramatically reduced capacity, as compared to wild-type and complemented strains, to induce NF-κB activation in HEK-TLR2 and THP-1 cells (Figures 5E and F) and IL-8 production in THP-1 and DCs (Figures 5G and H). Moreover, its uptake by THP-1 cells was decreased (Figure S2B). Δ*lspA* mutant reproducibly showed a defect stronger than that of Δ*pmmB* mutant (Figures 5E to H), further suggesting that lipoproteins play a predominant role as TLR2 ligands on *M. smegmatis* cell surface. Indeed, Δ*lspA* mutant produces non-mature lipoproteins, i.e. prolipoproteins, which remain sequestered to the cytosol and plasma membrane [40] and *per se* are devoid of TLR2 stimulating activity [22]. However, lipoproteins (more than 100 genes in *M. tuberculosis* genome [36]) have also physiological functions in the bacillus, most of them remaining unknown so far. Of note, one lipoprotein, lpqW, was recently implicated in the regulation of lipoglycan biosynthesis [42]. In this light, to determine whether prolipoprotein processing was required for proper lipoglycan biosynthesis, the latter were purified from the different strains and analyzed by SDS-PAGE. No quantitative differences were observed (not shown). However, interestingly both LAM and LM from Δ*lspA* mutant exhibited a lower apparent molecular weight (Figure 6B). This was confirmed by MALDI-TOF mass spectrometry analysis, which revealed molecular mass decreased by around 2 kDa, as compared to that of wild-type and complemented strains (Figure S4). Chemical analyses [29] indicated that this decrease in size resulted from a smaller mannan domain (not shown). So, these data indicate that *lspA* deletion in mycobacteria leads to an alteration of biosynthesis pathways not only of lipoproteins but also of the other family of TLR2 ligands, lipoglycans. Finally, it has been recently found that *M. tuberculosis* lipoprotein LprG is a glycolipid chaperone that increases the apparent potency of purified lipoglycans to stimulate TLR2 [43]. So, the strongly reduced potency of mycobacteria Δ*lspA* mutants to activate TLR2 most probably does not solely result from an altered stimulation by lipoprotein/lipopeptide agonists but rather by both lipoproteins/lipopeptides and lipoglycans.

### Conclusion

Innate immune detection of mycobacteria involves several PRRs that cooperate to mediate both uptake of the bacilli into host cells and activation of intracellular signaling cascades that signal the presence of the bacterial invader [44]. Among these receptors, TLR2 plays a key role in initiating the production of pro-inflammatory cytokines and chemokines that are crucial to eliciting the protective adaptative immune response [45]. We demonstrate here a direct correlation between the amount of lipoglycans in *M. smegmatis* cell envelope on one hand and the magnitude of innate immune signaling in HEK-TLR2 reporter cells, monocyte/macrophage THP-1 cell line and human dendritic cells, as revealed by NF-κB activation and IL-8 production, on the other hand. These data establish that lipoglycans are MAMPs contributing to innate immune detection of mycobacteria, *via* TLR2 among other PRRs. They also establish that ligands other than lipopeptides/lipoproteins can be sensed at physiological concentrations by TLR2. What are the precise cellular responses specifically associated to lipoglycan recognition remain to be investigated.

## Supporting Information

Figure S1 **List of primers used in this study.**
(DOC)

Figure S2 ***M. smegmatis*** **strains uptake by THP-1 cells.** Cells were infected for 1 h at 37°C with the various *M. smegmatis* strains at MOI of 50, extensively washed, lysed and plated onto agar for CFU counting. The results are expressed as the percentage of the inoculum being associated to the cells and are mean ± SD of triplicate wells and are representative of separate experiments using independent bacterial cultures and different MOI. **, P<0.01; ns, not significant.
(TIF)

Figure S3 **Expression of dendritic cell surface markers CD86 (A, B) and CD40 (C, D).** Cells were incubated overnight with the various *M. smegmatis* strains at MOI of 1.
(TIF)

Figure S4 **MALDI-TOF/MS analysis of LAM.** 0.5 µl of *M. smegmatis* wild-type (A), Δ*lspA* (B) and Δ*lspA*/CL (C) LAM solutions at 10 µg/µl were mixed with 0.5 µl of the matrix solution (10 µg/µl of 2,5-dihydroxybenzoic acid in ethanol/water, 1∶1, v/v) and analyzed by MALDI-TOF in the negative mode [29].
(TIF)
