# Supplementary material for: Lipoglycans Contribute to Innate Immune Detection of Mycobacteria
Source: PLoS One. 2011 Dec 2;6(12):e28476. doi: 10.1371/journal.pone.0028476 (PMC3229593; doi:10.1371/journal.pone.0028476)
Supplement: Figure S1 — List of primers used in this study. (DOC) [file pone.0028476.s001.doc]

| Name | Sequence |
| --- | --- |
| Rv3255cfw  Rv3255crev  Rv3257cfw  Rv3257crev  Rv3308fw  Rv3308rev  Rv3264cfw  Rv3264crev  FP:  RP:  FPB:  RPB:  RPI:  res1:  res2:  PMFP: PMRP:  3181A  3181B  3181C  3181D  3181I  3181J  res1b  res2b  kan1  kan2 | *M. smegmatis* recombinant strains  5’-AGCTAGGATATCCGGTGGAACTGCTACGTGGCGCGT-3’  5’-AGCTAGAAGCTTCAACCCGACGGTCGCCCT-3’  5’-AGCTAGTGGCCAGCATGTCTTGGCCCGCCGC-3’  5’-AGCTAGAAGCTTCGGTCCGGCCTTCGCAT-3’  5’-AGCTAGCCCGGGCCGTGACGCCAGAGAATTGGA-3’  5’-AGCTAGAAGCTTCCACCACCGCCGCA-3’  5’-AGCTAGGATATCAGTTGGCAACTCACCAAGT-3’  5’-AGCTAGAAGCTTAACGTCGGACGAGTAAC-3’  *M. smegmatis* Δ*pmmB* mutant  5’-ATGCGTTTAAACCGACCACGGCCTCACGCTGAGC-3’  5’-ATGCGTTTAAACGCGGTGGAGATCTGCCGGGCG -3’  5’-CTCTGCAGTTATCCCGACCTCGCCA-3’  5’-CACCGT TCCAGATCGGCGATCGAC-3’  5’-GGTTTCGTCGCCCGAGAGCATG-3  5’-GCTCTAGAGCAACCGTCCGAAATATTATAAA-3’  5’-GCTCTAGATCTCATAAAAATGTATCCTAAATCAAATATC-3’  5’- ATGCATATGACGTCGACCGCGACAACAGC-3’  5’- ATGCAAGCTTTCACCAGCC TTTCGCGGCGC -3’  *M. smegmatis* Δ*lspA* mutant  5’-TACCCGGCCACGATGTCGAC-3’  5’- GGATGCCCGAGGTGCTGATG-3’  5’-GCACGCAGATGGCGTTCGAC-3’  5’-AGGCACGCTTGAGCACGGTG-3’  5’- ACGGCATATGTGCGGACGGGTTCACGG-3’  5’- ACAAAAGCTTCTATGACGGCTCGGCCTGG-3’  5’-TCTAGAGCAACCGTCCGAAATA-3’  5’-GATCTCATAAAAATGTATCCTAAAT-3’  5’- AATCCATCTTGTTCAATCATGCG-3’  5’-TTCTATCGCCTTCTTGACGAG-3’ |
