# Supplementary figures and images for: Lipoglycans Contribute to Innate Immune Detection of Mycobacteria
Source: PLoS One. 2011 Dec 2;6(12):e28476. doi: 10.1371/journal.pone.0028476 (PMC3229593; doi:10.1371/journal.pone.0028476)

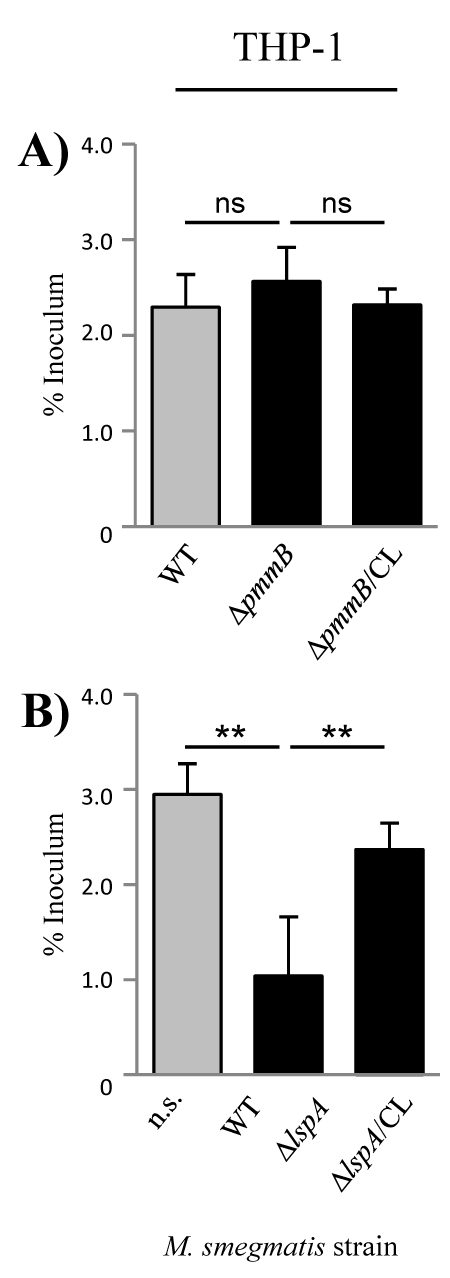

Supplement: Figure S2 — M. smegmatis strains uptake by THP-1 cells. Cells were infected for 1 h at 37°C with the various M. smegmatis strains at MOI of 50, extensively washed, lysed and plated onto agar for CFU counting. The results are expressed as the percentage of the inoculum being associated to the cells and are mean ± SD of triplicate wells and are representative of separate experiments using independent bacterial cultures and different MOI. **, P<0.01; ns, not significant. (TIF) [file pone.0028476.s002.tif]

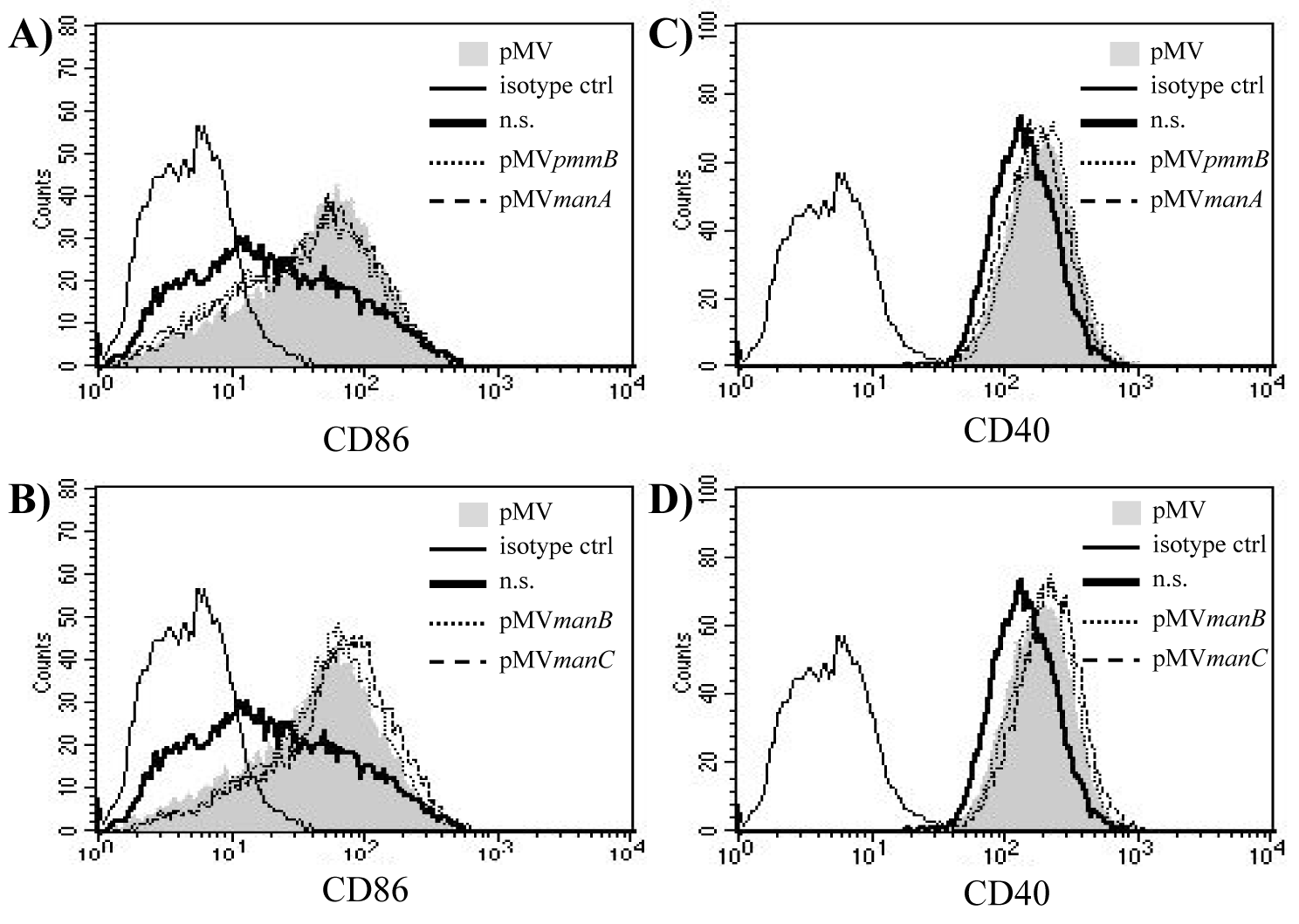

Supplement: Figure S3 — Expression of dendritic cell surface markers CD86 (A, B) and CD40 (C, D). Cells were incubated overnight with the various M. smegmatis strains at MOI of 1. (TIF) [file pone.0028476.s003.tif]

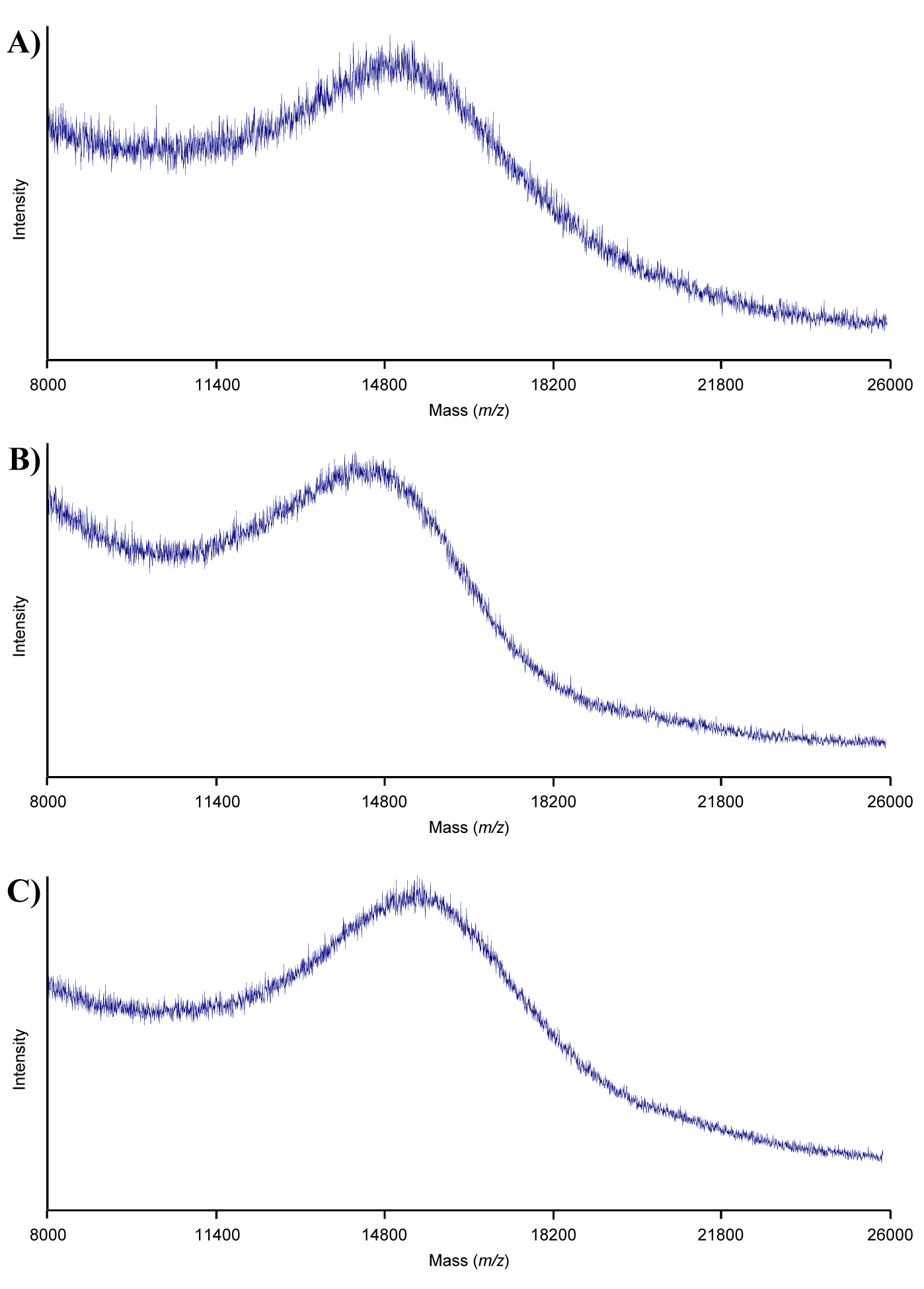

Supplement: Figure S4 — MALDI-TOF/MS analysis of LAM. 0.5 µl of M. smegmatis wild-type (A), ΔlspA (B) and ΔlspA/CL (C) LAM solutions at 10 µg/µl were mixed with 0.5 µl of the matrix solution (10 µg/µl of 2,5-dihydroxybenzoic acid in ethanol/water, 1∶1, v/v) and analyzed by MALDI-TOF in the negative mode [29]. (TIF) [file pone.0028476.s004.tif]
